# Automated vs. conventional ventilation in the ICU: a randomized controlled crossover trial comparing blood oxygen saturation during daily nursing procedures (*I-NURSING*)

**DOI:** 10.1186/s13054-020-03155-3

**Published:** 2020-07-22

**Authors:** Jonathan Chelly, Sandie Mazerand, Sebastien Jochmans, Claire-Marie Weyer, Franck Pourcine, Olivier Ellrodt, Nathalie Thieulot-Rolin, Jean Serbource-Goguel, Oumar Sy, Ly Van Phach Vong, Mehran Monchi

**Affiliations:** 1 Intensive Care Unit, Groupe Hospitalier Sud Ile de France, 270 avenue Marc Jacquet, 77000 Melun, France; 2 Clinical Research Unit, Groupe Hospitalier Sud Ile de France, 270 avenue Marc Jacquet, 77000 Melun, France

**Keywords:** Mechanical ventilation, Automated ventilation, Nursing procedure

## Abstract

**Background:**

Hypoxia is common during daily nursing procedures (DNPs) routinely performed on mechanically ventilated patients. The impact of automated ventilation on the incidence and severity of blood oxygen desaturation during DNPs remains unknown.

**Methods:**

A prospective randomized controlled crossover trial was carried out in a French intensive care unit to compare blood oxygen pulse saturation (SpO_2_) during DNPs performed on patients mechanically ventilated in automated and conventional ventilation modes (AV and CV, respectively). All patients with FiO_2_ ≤ 60% and without prone positioning or neuromuscular blocking agents were included. Patients underwent two DNPs on the same day using AV (INTELLiVENT-ASV®) and CV (volume control, biphasic positive airway pressure, or pressure support ventilation) in a randomized order. The primary outcome was the percentage of time spent with SpO_2_ in the acceptable range of 90–95% during the DNP.

**Results:**

Of the 265 included patients, 93% had been admitted for a medical pathology, the majority for acute respiratory failure (52%). There was no difference between the two periods in terms of DNP duration, sedation requirements, or ventilation parameters, but patients had more spontaneous breaths and lower peak airway pressures during the AV period (*p* <  0.001). The percentage of time spent with SpO_2_ in the acceptable range during DNPs was longer in the AV period than in the CV period (48 ± 37 vs. 43 ± 37, percentage of DNP period; *p* = 0.03). After adjustment, AV was associated with a higher number of DNPs carried out with SpO_2_ in the acceptable range (odds ratio, 1.82; 95% CI, 1.28 to 2.6; *p* = 0.001) and a lower incidence of blood oxygen desaturation ≤ 85% (adjusted odds ratio, 0.50; 95% CI, 0.30 to 0.85; *p* = 0.01).

**Conclusion:**

AV appears to reduce the incidence and severity of blood oxygen desaturation during daily nursing procedures (DNPs) in comparison to CV.

**Trial registration:**

This study was registered in clinical-trial.gov (NCT03176329) in June 2017.

**Graphical abstract:**

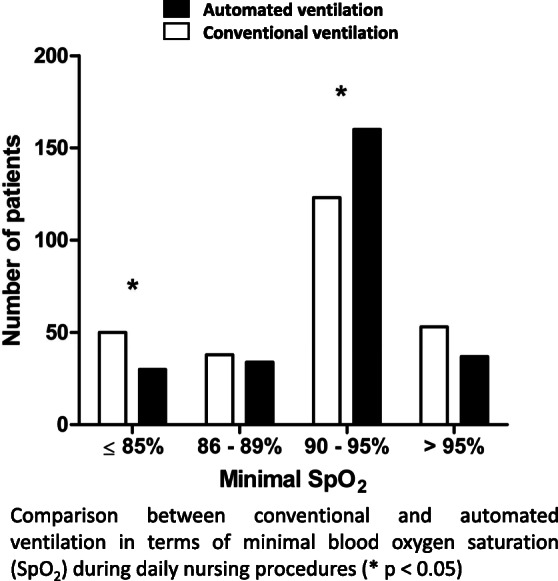

## Background

Daily nursing procedures (DNPs) are routinely performed several times per day in the intensive care unit (ICU) and are crucial for patients’ hygiene and rehabilitation, and to prevent/treat complications due to immobilization [1–4]. However, these DNPs induce physiological changes with potential adverse effects, especially in patients undergoing mechanical ventilation (MV) [5–9]. Respiratory events, in particular oxygen desaturation, are often observed during DNPs but are not well documented [10]. Although the potential adverse effects may be serious (e.g., severe hypoxemia and cardiac arrest), these events are often downplayed, considered as a normal part of DNPs, or ignored in interventional studies regarding MV in ICU patients [10]. As suggested by previous works, protocols should be developed to prevent such respiratory events [11, 12]. INTELLiVENT-ASV® (Hamilton Medical, Bonaduz, Switzerland) is an automated ventilation mode (AV) that automatically adjusts ventilation and oxygenation settings to keep end-tidal CO_2_ (PetCO_2_) and SpO_2_ in target ranges set by the clinician [13]. Briefly, minute volume is adjusted according to PetCO_2_ information or respiratory rate in passive or spontaneously breathing patients respectively and FiO2 and PEEP are adjusted according to blood oxygen pulse saturation (SpO_2_) information. The safety, feasibility, and efficacy of this mode have been demonstrated with promising results that show a reduction in both the number of manual interventions needed and the incidence of blood oxygen desaturation for various patient conditions, including acute respiratory failure, post-cardiac surgery, and weaning from MV [13–20]. To our knowledge, however, no study has assessed the impact of AV on the incidence of respiratory events during DNPs. The aim of our study was to compare the incidence and severity of blood oxygen desaturation during DNPs performed on patients ventilated in AV and in conventional ventilation mode (CV).

## Methods

### Settings and patients

This single-center randomized controlled crossover study was conducted from September 2016 to March 2018 in a 22-bed, mixed ICU of a French tertiary center. All patients of both sexes mechanically ventilated for at least 48 h with a fraction of inspired oxygen (FiO_2_) ≤ 60% were included. Exclusion criteria were prone positioning, use of neuromuscular blocking agents, age < 18 years old, pregnant women, patients with a contraindication to AV (delirium, broncho-pleural fistula, respiratory drive disorder such as Cheyne-Stokes breathing), and patients with a low-quality measurement for SpO_2_. The study was initiated and supported by the Groupe Hospitalier Sud Ile de France (Melun, France). The study protocol was approved on the 13th of September 2016 by the ethical committee (Comité de Protection des Personnes Ile de France VI) and registered in ClinicalTrials.gov (NCT03176329). Patients or their next-of-kin gave their informed consent before randomization.

### Study protocol and data collection

All included patients were ventilated using a HAMILTON-S1 ventilator (Hamilton Medical, Bonaduz, Switzerland) and SpO_2_ was monitored using a dedicated sensor (Masimo SET®, Masimo Corporation, Irvine, USA) connected to the patient’s monitor (Beneview T8®, Mindray, Shenzhen, China). After randomization, each patient underwent two DNPs on the same day, with 6 h between the two. One was performed in CV and the other in AV in a randomized order defined at inclusion. DNPs were performed by two nurses and included a bundle of care covering patient hygiene (bathing, change of bed linen), mobilization (repositioning), ventilator-associated pneumonia prevention (oral hygiene care, subglottic secretion drainage, adjustment of endotracheal tube cuff pressure), and pressure ulcer prevention and treatment (massage of back and pressure points). All the patient’s monitoring and ventilation parameters, including heart rate, mean arterial pressure, SpO_2_, FiO_2_, expiratory tidal volume, total and spontaneous respiratory rate, positive end-expiratory pressure (PEEP), and inspiratory and mean airway pressure, were automatically recorded every minute during the DNP using Evolucare Intensive 6.4® (Evolucare Technologies, Villers-Bretonneux, France). Arterial blood gas samples were performed 5 min before each DNP to determine the PaO_2_/FiO_2_ ratio. All events that occurred during the DNPs, such as blood oxygen desaturation, change of ventilation mode, accidental disconnection of the ventilator, the need for manual ventilation, activation of an oxygen bypass, or endotracheal suctioning, were also reported by the nurse in charge.

### Ventilation settings

At least 30 min before each of the two DNPs, the attending physician set the MV mode according to the randomized order. For the CV period, the mode and ventilator settings were selected by the attending physician according to patient’s pathologies and conditions: either volume control (VC), biphasic positive airway pressure (BIPAP), or pressure-support ventilation (PSV). In VC mode, the tidal volume (*V*_*T*_) was set below 7 mL/kg predicted body weight (PBW) for acute respiratory distress syndrome, below 9 mL/kg PBW for subjects with normal lungs, and below 11 mL/kg PBW for subjects with chronic obstructive pulmonary disease. In BIPAP and PSV, inspiratory pressure and pressure support were set according to the same *V*_*T*_ limits as in VC. Plateau pressure was limited to 30 cmH_2_O, while positive end-expiratory pressure (PEEP) and FiO_2_ were set to maintain SpO_2_ before the DNPs at between 94 and 98% [21, 22]. During the DNPs, nurses were responsible for maintaining SpO_2_ within an acceptable range of 90–95% by adjusting the FiO_2_ setting. The basic principles of INTELLiVENT-ASV® are detailed as previously described [13] in the Online supplemental content 1. Before the DNP in AV, automated FiO_2_ and PEEP controllers were set by the attending physician with a lower limit for SpO_2_ at 90% and a PEEP limited to 5–15 cmH_2_O. The high limit for airway pressure was set at a maximum of 30 cmH_2_O. For both DNPs, alarm limits were set by the clinicians. In the case of major blood oxygen desaturation (SpO_2_ ≤ 85% according to guidelines [22]), the nurse in charge was required to apply a specific protocol (see Online supplemental content 2) and a physician was always present in the ICU in case a problem persisted. Sedation infusion, inspiratory trigger, pressure rise, expiratory trigger, and ventilator circuit were the same in both periods.

### Study outcomes

The primary outcome was the time spent with SpO_2_ values of 90–95% (considered to be the acceptable SpO_2_ range during the DNP). Secondary outcomes were as follows: Incidence of SpO_2_ in the acceptable range during the DNP; mean, minimum, and maximum SpO_2_ during the DNP (SpO_2 mean_, SpO_2 min_, and SpO_2 max_, respectively); incidence and time spent with SpO_2_ lower than 90%; incidence and time spent with SpO_2_ lower than 85%; and time spent with FiO_2_ at 100%. The safety outcome parameters were the occurrence of major adverse events (accidental endotracheal tube removal, bradycardia lower than 40 bpm, or cardiac arrest) during the DNP. Two interim analyses for primary and safety outcome parameters were planned after 90 and 180 patients were enrolled.

### Statistical analysis

Based on previous studies on DNPs [9, 10] and retrospective data collected in our institution, we estimated that patients spent 40% of the DNP duration with SpO_2_ between 90 and 95%. We calculated a sample size of 267 patients by group to detect a 15% increase in the primary outcome for DNPs performed in AV as compared to CV (2-sided α = 0.05; power 80%). Continuous variables are expressed by mean ± standard deviation and nominal variables as *n* (%). Continuous variables were compared using the non-parametric Wilcoxon test and nominal variables were compared using Fisher’s exact test. After a univariate analysis to assess all risk factors for primary and secondary outcomes, a multivariate analysis was performed including all univariate factors with *p* <  0.15. Differences were considered significant where *p* <  0.05. All calculations were performed using SPSS Statistics V20® (IBM, New York, USA).

## Results

**Fig. 1:**
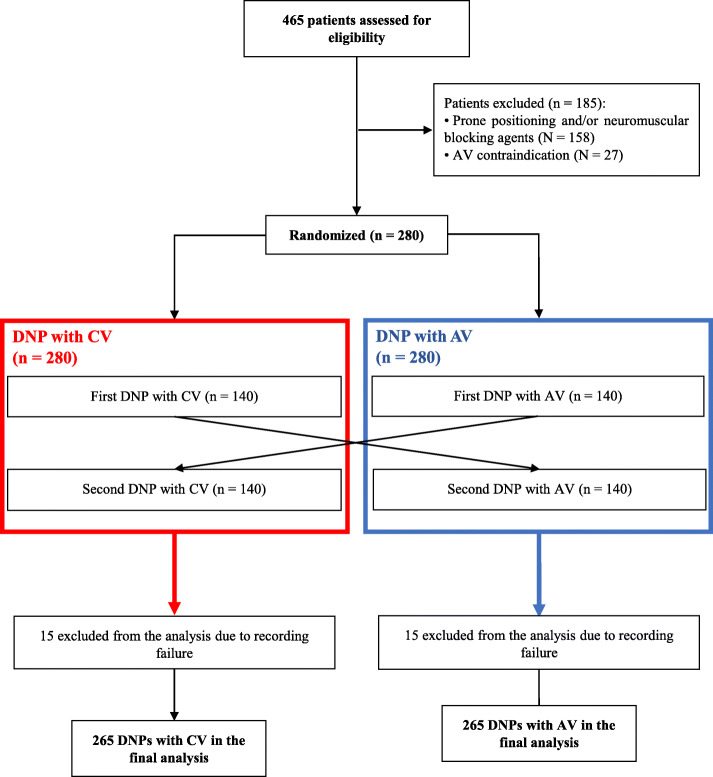
*I-NURSING* trial flow diagram (DNP, daily nursing procedures; CV, conventional ventilation; AV, automated ventilation)

There were no safety issues that required premature interruption. Among the 465 patients assessed for eligibility, 185 were excluded, leaving 280 for inclusion (Fig. 1). Fifteen patients in both periods were subsequently excluded from the analysis due to recording failure, resulting in 265 patients with one DNP in each period for the final analysis.

### Patient characteristics

Baseline characteristics of the overall cohort are detailed in Table 1. Patients were primarily admitted for a medical pathology (92%) and half were intubated for acute respiratory failure (52%). The mean time of MV before inclusion and the total MV duration were 4 ± 4 and 11 ± 8 days, respectively. Of the 265 patients, 201 (76%) were successfully weaned from MV and 74 (28%) died before ICU discharge. The interval between both DNPs was 406 ± 118 min. Before the DNP, SpO_2_ was significantly lower in AV than in CV (95 ± 3% vs. 96 ± 3%, respectively; *p* <  0.001), whereas PaO_2_/FiO_2_ and SpO_2_/FiO_2_ were similar (*p* = 0.10 and 0.49, respectively). DNP duration varied from 2 to 55 min in the overall cohort. As detailed in Table 2, DNP duration and patient sedation levels were similar during both periods, as were hemodynamic parameters, tidal volume, total breathing rate, and PEEP level. During the CV period, BIPAP mode was largely used (87%) and patients had a significantly lower spontaneous breathing rate than during the AV period (14 ± 11 vs. 22 ± 12 breath/min, respectively; *p* <  0.001). Patients had a lower peak airway pressure (25 ± 7 vs. 27 ± 6 cmH_2_O; *p* <  0.001) and mean airway pressure (14 ± 4 vs. 15 ± 4 cmH2O; *p* <  0.001) during the AV period than during the CV period.

**Table 1:** Patients’ baseline characteristics at inclusion

Characteristics	Overall cohort *n* = 265
Age—years	64 ± 14 (62–66)
Men/women—*n* (%)	172 (65%) / 93 (35%)
Body mass index—kg/m^2^	31 ± 15 (29–32)
Chronic disease—*n* (%)	
COPD	55 (21%)
Chronic respiratory failure	26 (10%)
Chronic heart failure	34 (13%)
SAPS-2 at ICU admission	59 ± 19 (56–61)
SOFA at inclusion	9 ± 3 (8–9)
Reason for ICU admission—*n* (%)	
Medical	245 (92%)
Surgical	20 (8%)
Acute organ failure at inclusion—*n* (%)	178 (67%)
Hemodynamic	122 (46%)
Kidney	57 (22%)
Neurologic	42 (16%)
Heart	25 (9%)
Liver	22 (8%)
Hematologic	9 (3%)
Main reason for MV—*n* (%)	
Acute respiratory failure	138 (52%)
Coma	67 (25%)
Cardiac arrest	19 (7%)
Sepsis	15 (6%)
Other	24 (9%)
Unknown	2 (1%)
MV duration before inclusion—days	4 ± 4 (4–5)
Chest radiograph opacities—quadrants	2 ± 1 (1–2)

Categorical variables are expressed as *n* (%) and continuous variables as mean ± standard deviation (95% confidence interval)

*COPD* chronic obstructive pulmonary disease, *SAPS-2* Simplified Acute Physiological Score 2, *SOFA* Sepsis-Related Organ Failure Assessment, *ICU* intensive care unit, *MV* mechanical ventilation

**Table 2:** General, hemodynamic and ventilation parameters during daily nursing procedure (*DNPs*) according to ventilation mode (*CV* conventional ventilation, *AV* automated ventilation)

Parameters	CV period *n* = 265	AV period *n* = 265	*p*
DNP parameters			
DNPs duration—min	12 ± 6 (11–13)	12 ± 8 (11–13)	0.95
Ramsay score	4 ± 2 (4–4)	4 ± 1 (4–4)	0.35
Oxygenation before DNPs			
SpO_2_—%*	96 ± 3 (95–96)	95 ± 3 (95–95)	< 0.001
SpO_2_/FiO_2_*	320 ± 79 (310–329)	317 ± 87 (306–327)	0.49
PaO_2_/FiO_2_**	268 ± 95 (255–282)	266 ± 92 (253–280)	0.10
Hemodynamics during DNPs			
Heart rate—bpm †	98 ± 19 (95–100)	98 ± 20 (96–101)	0.32
Mean arterial pressure—mmHg^†^	89 ± 14 (87–90)	88 ± 15 (86–90)	0.56
Ventilation during DNPs			
Mode used in the CV period—*n* (%)			
Biphasic positive airway pressure	230 (87%)	–	–
Pressure support ventilation	23 (9%)	–	–
Volume controlled	12 (4%)	–	–
Tidal volume—mL/kg of PBW^†^	10 ± 3 (10–10)	10 ± 2 (10–10)	0.40
Total RR—breath/min^†^	28 ± 9 (27–29)	27 ± 8 (26–28)	0.79
Spontaneous RR—breath/min^†^	14 ± 11 (13–15)	22 ± 12 (21–24)	< 0.001
Passive ventilation—*n* (%)***	35 (13%)	9 (3%)	< 0.001
Peak airway pressure—cmH_2_O^†^	27 ± 6 (26–27)	25 ± 7 (24–26)	< 0.001
Mean airway pressure—cmH_2_O^†^	15 ± 4 (14–15)	14 ± 4 (14–15)	< 0.001
PEEP—cmH_2_O^†^	9 ± 3 (9–10)	9 ± 3 (9–10)	0.07

Categorical variables are expressed as *n* (%) and continuous variables as mean ± standard deviation (95% confidence interval)

*PBW* predicted body weight, *RR* respiratory rate, *PetCO*_*2*_ end-tidal CO_2_ partial pressure, *PEEP* positive end-expiratory pressure

*Measured the minute before starting the DNP

**Measured 5 min before starting the DNP

***Defined as no spontaneous breathing detected by the ventilator

^†^Mean of the overall parameters monitored every minute during the DNP

### Endpoints

**Fig. 2:**
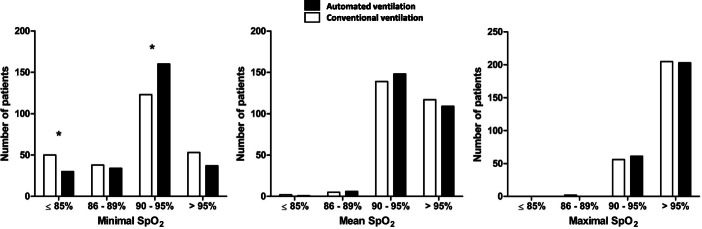
Comparison between conventional and automated ventilation in terms of minimal, mean, and maximal blood oxygen saturation (SpO_2_) during daily nursing procedures (*p <  0.05)

Data for the primary and secondary endpoints are provided in Table 3. Patients spent significantly more time in the acceptable SpO_2_ range during the AV period (48 ± 37 vs. 43 ± 37% of DNP period; *p* = 0.03). In 160 patients (60%), SpO_2_ was in the acceptable range during the AV period as compared to 123 patients (46%) during the CV period (*p* = 0.001). After adjustment for confounding factors, AV was associated with a greater number of DNPs performed with SpO_2_ in the acceptable range (odds ratio [OR], 1.82; 95% confidence interval [CI], 1.28 to 2.6; *p* = 0.001; see Online supplemental content 3). In the overall cohort, blood oxygen desaturation to levels < 90% and ≤ 85% occurred in 161 (30%) and 80 (15%) patients, respectively. Incidences of blood oxygen desaturation to lower than 90% were less frequent during the AV period than during the CV period (69 [26%] vs. 92 [35%], episodes > 1 min; *p* = 0.03) and were also shorter (5 ± 12 vs. 6 ± 11, % of DNP period; *p* = 0.02). Incidences of major blood oxygen desaturation (≤ 85%) were less frequent during the AV period than during the CV period (30 [11%] vs. 50 [19%], episodes > 1 min; *p* = 0.02) and were also shorter (2 ± 6 vs 3 ± 8, percentage of DNP period; *p* = 0.03). After adjustment for confounding factors, AV was associated with a lower incidence of blood oxygen desaturation ≤ 85% during DNPs (OR, 0.50; 95% CI, 0.30 to 0.85; *p* = 0.01; see Online supplemental content 4). As shown in Fig. 2, more patients had SpO_2min_ in the optimal range during the AV period (*p* = 0.02), while more patients had the SpO_2min_ ≤ 85% during the CV period (*p* = 0.002). There was no difference between the two periods for the other secondary endpoints (Table 3).

**Table 3:** Primary and secondary outcome parameters according to ventilation mode (*CV* conventional ventilation, *AV* automated ventilation)

Outcome parameters	CV period *n* = 265	AV period *n* = 265	*p*
Primary outcome			
Time spent with SpO_2_ in the acceptable range—% of DNP duration*	43 ± 37 (38–47)	48 ± 37 (43–52)	0.03
Secondary outcome			
Patients with SpO_2_ in the acceptable range during DNP—*n* (%)*	123 (46%)	160 (60%)	0.001
Patients with at least one episode of:			
SpO_2_ ≤ 85%**—*n* (%)	50 (19%)	30 (11%)	0.02
SpO_2_ < 90%**—*n* (%)	92 (35%)	69 (26%)	0.03
Time spent with:			
SpO_2_ ≤ 85%—% of DNP duration	3 ± 8 (2–4)	2 ± 6 (1–2)	0.03
SpO_2_ < 90%—% of DNP duration	6 ± 11 (5–7)	5 ± 12 (3–6)	0.02
FiO_2_ 100%—% of DNP duration	6 ± 12 (4–7)	4 ± 10 (3–6)	0.09
SpO_2 mean_—%	95 ± 3 (95–95)	95 ± 3 (95–95)	0.81
SpO_2 min_—%	91 ± 6 (90–91)	91 ± 8 (90–92)	0.20
SpO_2 max_—%	97 ± 2 (97–98)	97 ± 2 (97–98)	0.63

Categorical variables are expressed as *n* (%) and continuous variables as mean ± standard deviation (95% confidence interval)

*DNP* daily nursing procedure, *SpO*_*2*_ blood oxygen pulse saturation, *FiO*_*2*_ fraction of inspired oxygen, *SpO*_*2 mean*_ mean SpO_2_ during DNP, *SpO*_*2 min*_ minimal SpO_2_ recorded during DNP, *SpO*_*2 max*_ maximal SpO_2_ recorded during DNP

*SpO_2_ acceptable range was ≥ 90 and ≤ 95%

**For more than 1 min

### Nurse/physician/ventilator interventions, safety, and major adverse events

All the unplanned interventions performed by a nurse or physician during DNPs are detailed in the Online supplement content 5. A change of ventilation mode occurred in only one patient in each period, and two patients required manual ventilation in the CV period. Patients required fewer manual activations of an oxygen bypass during the AV period than during the CV period (41 [15%] vs. 69 [26%]; *p* = 0.004) and less endotracheal suctioning per DNP (1 ± 0 vs 1 ± 1; *p* = 0.03). PEEP was manually increased in two patients (1%) during the CV period and automatically increased by the ventilator in 53 patients (20%) during the AV period. With the exception of one episode of bradycardia (< 40 bpm) in one patient during the AV period, no major adverse events occurred during DNPs in either period. Accidental disconnection of the ventilator occurred in 18 (7%) and 16 (6%) patients during the AV and CV periods, respectively (*p* = 0.9).

## Discussion

This is the first study to test the ability of AV to reduce the occurrence of oxygen desaturation during DNPs routinely performed in ICU patients undergoing MV. In this randomized crossover trial, the use of AV (in this case INTELLiVENT®-ASV®) was superior to CV with respect to maintaining SpO2 within an acceptable range and reducing the incidence and severity of oxygen desaturation.

Although ICU nurses and physicians frequently observe blood oxygen desaturation relating to DNPs in their daily practice, these respiratory events remain poorly documented. In a cohort of 53 ICU patients (including 45% under MV), De Jong et al. observed blood oxygen desaturation ≤ 90% and ventilatory distress (severe patient-ventilator asynchrony, nonstop coughing, impossible ventilation, and/or tachypnea) in 10 and 13%, respectively, of the 184 DNPs performed [9]. In a prospective study on 16 ICU patients undergoing MV, 668 nursing procedures were observed and blood oxygen desaturation ≤ 90% was the most frequent adverse event described, representing 29% of the overall major physiological changes reported by the authors [10]. For our overall cohort, we reported blood oxygen desaturation < 90 and ≤ 85% during DNPs in 30 and 15%, respectively.

Various physiological changes may be implicated by the occurrence of blood oxygen desaturation during DNPs. Patient mobilization is one of the most important, and in particular lateralization, which can induce a decrease in lung compliance, alveolar derecruitment, mobilization of respiratory-tract secretions, airway irritations and coughing, ventilator-patient asynchrony [10, 23–26], and an increase in oxygen consumption [5, 6]. All those physiological events could be induced by mobilization itself and/or the stress response associated with pain [10, 27–29].

The impact on patient outcomes of DNPs and their related adverse events remains unclear. Previous studies have suggested that early mobilization of the patient would be associated with a greater chance of achieving rehabilitation objectives in the ICU setting [1–4]. De Jong et al. observed an incidence of cardiac arrest in 1% of the DNPs performed, while we did not report any incidence of cardiac arrest or death related to DNPs in our overall cohort.

Our study suggests AV may have a protective effect when compared to CV in terms of SpO_2_ values and the incidence and severity of blood oxygen desaturation during DNPs. A prospective randomized controlled study of 60 post-cardiac surgery patients showed that in comparison to CV, INTELLiVENT-ASV® significantly reduces the percentage of time, as well as the total duration and number of episodes per patient of ventilation parameters (including tidal volume, EtCO_2_, plateau pressure, and SpO_2_) being within a “not acceptable” zone [14]. During the weaning period in 16 ICU patients, INTELLiVENT-ASV® improved the PaO_2_/FiO_2_ ratio compared to PSV [15]. In accordance with our results, another randomized trial including 80 ICU patients showed that INTELLiVENT-ASV® was superior to pressure assist-control and PSV for maintaining SpO_2_ in an optimal range defined by the authors as between 92 and 96% [18].

The positive results of AV on the incidence of blood oxygen desaturation may be explained by many factors: INTELLiVENT-ASV® continuously and quickly adapts oxygenation, increasing PEEP and FiO_2_ when SpO_2_ decreases, but also by automatically decreasing PEEP and FiO_2_ when SpO_2_ is supranormal. In contrast, nurses and physicians are not able to adjust FiO_2_ every time while they are providing care, especially during mobilization of a patient. As suggested by our results, the need for endotracheal suctioning during DNPs seems less frequent in AV, which could be interpreted as the cause or the consequence of a lower incidence of blood oxygen desaturation.

### Limitations of the study

Several factors may limit the interpretation of our data. First, this was a single-center study in a single-blinded design, carried out in an ICU staffed by nurses and physicians considered as advanced users of AV. However, previous studies have consistently reported on the efficacy and safety advantages of using AV over CV [13–20]. Second, patients in unstable respiratory conditions, such as high FiO2 > 60% with or without the use of neuromuscular blocking agents and/or with prone positioning, were excluded from the study. In our ICU, as is general practice in many ICUs, DNPs in patients with unstable respiratory conditions are delayed until the patient’s condition improves or performed with FiO_2_ set at 100% by default. Third, we should have systematically assessed and prevented pain related to patient’s care. Indeed, the incidence of respiratory events decrease significantly with the application of an analgesic protocol before and during DNP, as previously described by De Jong et Al. Future studies on DNP should take pain prevention and treatment into account [9]. Moreover, we cannot draw any conclusions with respect to a protective or harmful effect of AV in terms of ventilator-induced lung injury (VILI) during DNPs. Tidal volumes were higher than the initial setting for both periods, probably due to an increase in the patient’s ventilatory drive during mobilization (induced by stress, pain, etc.). However, inspiratory pressure was lower and spontaneous breathing was higher during DNPs in AV. Future studies are needed to assess mechanical power and the risk of VILI during DNPs [30]. Fourth, we may have underestimated the incidence of short oxygen desaturation (< 1 min) because we have not performed a breath by breath monitoring. Fifth, the accuracy of SpO_2_ measurements remains controversial, particularly in ICU patients with acute organ failure, as previously observed [31]. However, blood gas samples are not easy to perform during DNP and SpO_2_ represents the only parameters to assess oxygenation at bedside during this procedure. Finally, although we found a significant difference in the primary outcome in favor of AV during DNPs, the clinical impact remains unknown. Further studies are warranted to confirm our results and to assess the real impact on patient outcomes and management.

## Conclusion

AV used during DNPs routinely performed on ICU patients undergoing MV appears to be superior to CV in maintaining SpO_2_ within an acceptable range and reducing the incidence and severity of desaturation, with more spontaneous breathing and lower peak and mean airway pressure.

## Supplementary information

**Additional file 1: Online supplemental content 1.** Basic principles of INTELLiVENT-ASV®. **Supplemental content 2.** Protocol for nurses in case of major blood oxygen desaturation (SpO_2_ ≤ 85%) during the daily nursing procedure (DNP). **Supplemental content 3.** Multivariate logistic regression test for SpO_2_ in the acceptable range (between 90% and 95%) during the daily nursing procedure (*DNP*). **Supplemental content 4.** Multivariate logistic regression test of risk factors for occurrence of at least one major oxygen desaturation (SpO_2_ ≤ 85%) during the daily nursing procedure (*DNP*). **Supplemental content 5.** Nurse/physician interventions during the daily nursing procedure (*DNP*) according to ventilation mode (*CV* conventional ventilation; *AV* automated ventilation).

## Data Availability

The dataset used and/or analyzed during the current study are available from the corresponding author on reasonable request.

## Abbreviations

AV: Automated ventilation mode
BIPAP: Biphasic positive airway pressure
CI: Confidence interval
CV: Conventional ventilation mode
DNP: Daily nursing procedure
FiO_2_: Fraction of inspired oxygen
ICU: Intensive care unit
MV: Mechanical ventilation
PEEP: Positive end-expiratory pressure
PetCO_2_: End-tidal CO_2_ partial pressure
PSV: Pressure support ventilation
SpO_2_: Blood oxygen pulse saturation
VC: Volume control
VILI: Ventilator-induced lung injury
