# Supplementary material for: Automated vs. conventional ventilation in the ICU: a randomized controlled crossover trial comparing blood oxygen saturation during daily nursing procedures (I-NURSING)
Source: Crit Care. 2020 Jul 22;24:453. doi: 10.1186/s13054-020-03155-3 (PMC7374079; doi:10.1186/s13054-020-03155-3)
Supplement: Supplementary file 1 — Additional file 1: Online supplemental content 1. Basic principles of INTELLiVENT-ASV®. Supplemental content 2. Protocol for nurses in case of major blood oxygen desaturation (SpO2 ≤ 85%) during the daily nursing procedure (DNP). Supplemental content 3. Multivariate logistic regression test for SpO2 in the acceptable range (between 90% and 95%) during the daily nursing procedure (DNP). Supplemental content 4. Multivariate logistic regression test of risk factors for occurrence of at least one major oxygen desaturation (SpO2 ≤ 85%) during the daily nursing procedure (DNP). Supplemental content 5. Nurse/physician interventions during the daily nursing procedure (DNP) according to ventilation mode (CV conventional ventilation; AV automated ventilation). [file 13054_2020_3155_MOESM1_ESM.docx]

**Automated vs. conventional ventilation in the ICU: A randomized controlled crossover trial comparing blood oxygen saturation during daily nursing procedures (*I-NURSING*)**

Jonathan Chelly, Sandie Mazerand, Sebastien Jochmans, Claire-Marie Weyer, Franck Pourcine, Olivier Ellrodt, Nathalie Thieulot-Rolin, Jean Serbource-Goguel, Oumar Sy, Ly Van Phach Vong, Mehran Monchi

***ONLINE SUPPLEMENTAL CONTENT***

**Online supplemental content – 1. Basic principles of INTELLiVENT-ASV^®^**

INTELLiVENT-ASV**^®^** is a fully closed loop ventilation mode, which manages both the ventilation and oxygenation settings automatically using the data supplied by an end-tidal CO_2_ (PetCO_2_) and a blood oxygen pulse saturation (SpO_2_) sensor. The goal is to reach the ventilation and oxygenation targets set by the clinician.

The user sets patient’s gender and height, and patient’s condition between normal lungs, acute respiratory distress syndrome (ARDS), chronic hypercapnia, and brain injury. By selecting patient’s condition, the algorithm proposes different default PetCO_2_ and SpO_2_ target ranges. In passive patients, the ventilation controller adjusts the target minute volume (MinVol) according to the measured PetCO_2_ and target PetCO_2_ set by the clinician. In spontaneously breathing patients, the ventilation controller adjusts the target MinVol to keep the patient’s respiratory rate in an acceptable range. For any given target MinVol, the INTELLiVENT-ASV**^®^** controller determines the optimal tidal volume-respiratory rate combination and delivers either an adaptive pressure-controlled breath in passive patients, or an adaptive pressure-supported breath in spontaneously breathing patients.

The oxygenation controller adjusts the fraction of inspired oxygen (FiO_2_) and the positive end-expiratory pressure (PEEP) to keep the patient’s SpO_2_ within the target SpO_2_ range set by the clinician. A PEEP/FiO_2_ table derived from the ARDS network publications is used to determine the combination of PEEP and FiO_2_.

**Supplemental content – 2. Protocol for nurses in case of major blood oxygen desaturation (SpO_2_ ≤ 85%) during the daily nursing procedure (DNP).**

For DNPs performed in conventional ventilation mode:

1. Stop the DNP and place the patient in a supine position.
2. Increase FiO_2_ to reach SpO_2_ ≥ 90 and ≤ 95%. If SpO_2_ decreases to ≤ 75%, activate the oxygen bypass on the ventilator (FiO_2_ will be set at 100% for 2 min).
3. Check the SpO_2_ shape on the monitor and position of the SpO_2_ sensor on the patient.
4. Carry out endotracheal suctioning in the case of tube obstruction due to tracheal secretions.
5. Resume the DNP when SpO_2_ ≥ 90% by adjusting FiO_2_.
6. In the case of persistent SpO_2_ < 90%, keep FiO_2_ at 100% and call the attending physician.

For DNPs performed in automated ventilation mode:

1. Stop the DNP and place the patient in a supine position.
2. If the ventilator is not increasing FiO_2_ and/or SpO_2_ decreases to ≤ 75% with FiO_2_ < 100%, activate the oxygen bypass on the ventilator (FiO_2_ will be set at 100% for 2 min).
3. Check the SpO_2_ shape on the monitor and position of the SpO_2_ sensor on the patient.
4. Carry out endotracheal suctioning in the case of tube obstruction due to tracheal secretions.
5. Resume the DNP when SpO_2_ ≥ 90%.
6. In the case of persistent SpO_2_ < 90%, set FiO_2_ manually to 100% (by disabling the automated FiO_2_ controller) and call the attending physician.

**Supplemental content – 3.** Multivariate logistic regression test for SpO_2_ in the acceptable range (between 90% and 95%) during the daily nursing procedure (*DNP*).

| Parameters | 90 ≤ SpO_2_ ≤ 95% | | p  univariate | OR (95% CI) | p  multivariate |
| --- | --- | --- | --- | --- | --- |
|  | **Yes**  **n= 283** | **No**  **n = 247** |  |  |  |
| Age | 65 ± 14 | 63 ± 15 | 0.134 | 1.01 (0.99 – 1.03) | 0.06 |
| DNP duration (min) | 12 ± 7 | 13 ± 8 | 0.04 | 0.99 (0.96 – 1.01) | 0.37 |
| Heart rate (bpm) | 96 ± 20 | 100 ± 19 | 0.05 | 0.99 (0.99 – 1.00) | 0.20 |
| COPD | 66 (23%) | 44 (18%) | 0.13 | 1.47 (0.94 – 2.29) | 0.09 |
| Chronic heart failure | 29 (10%) | 39 (16%) | 0.07 | 0.52 (0.3 – 0.9) | 0.02 |
| Acute organ failure | 181 (64%) | 175 (71%) | 0.09 | 0.73 (0.5 – 1.08) | 0.11 |
| Ventilator accidental disconnection | 12 (4%) | 22 (9%) | 0.03 | 0.46 (0.22 – 0.99) | 0.05 |
| DNP using the AV | 160 (57%) | 105 (43%) | 0.002 | 1.82 (1.28 – 2.6) | 0.001 |
| Categorical variables are expressed as n (%) and continuous variables as mean ± standard deviation. Each parameter was included in the multivariate logistic regression if p < 0.15 in the univariate analysis. Odds ratio (*OR*) are expressed with their 95% confidence interval (*CI*). Hosmer and Lemeshow goodness of fit test *p* = 0.5; *COPD* chronic obstructive pulmonary disease; *AV* automated ventilation | | | | | |

**Supplemental content – 4.** Multivariate logistic regression test of risk factors for occurrence of at least one major oxygen desaturation (SpO_2_ ≤ 85%) during the daily nursing procedure (*DNP*)

| Parameters | SpO_2_ ≤ 85% | | p  univariate | OR (95%CI) | p  multivariate |
| --- | --- | --- | --- | --- | --- |
|  | **Yes**  **n = 80** | **No**  **n = 447** |  |  |  |
| SOFA | 10 ± 3 | 9 ± 3 | 0.005 | 1.11 (1.00 – 1.22) | 0.05 |
| MV duration before inclusion (days) | 3 ± 3 | 4 ± 4 | 0.07 | 0.94 (0.87 – 1.01) | 0.11 |
| Chest radiograph opacities (quadrants) | 2 ± 1 | 1 ± 1 | 0.04 | 1.23 (0.99 – 1.51) | 0.05 |
| DNP duration (min) | 15 ± 9 | 12 ± 7 | 0.002 | 1.05 (1.01 – 1.08) | 0.005 |
| Chronic lung failure | 12 (15) | 40 (9) | 0.10 | 1.14 (0.51 – 2.56) | 0.75 |
| Chronic heart failure | 19 (24) | 48 (11) | 0.003 | 2.16 (1.09 – 4.24) | 0.03 |
| Acute organ failure | 63 (79) | 292 (65) | 0.02 | 1.47 (0.76 – 2.85) | 0.25 |
| Endotracheal suctioning required during DNP | 38 (48) | 157 (35) | 0.04 | 1.57 (0.92 – 2.68) | 0.1 |
| DNP using the AV | 30 (38) | 234 (52) | 0.02 | 0.50 (0.30 – 0.85) | 0.01 |
| Categorical variables are expressed as n (%) and continuous variables as mean ± standard deviation. Each parameter was included in the multivariate logistic regression if p < 0.15 in the univariate analysis. Odds ratio (*OR*) are expressed with their 95% confidence interval (*CI*). Hosmer and Lemeshow goodness of fit test *p* = 0.06. *SOFA* sepsis-related organ failure assessment; *MV* mechanical ventilation; *PetCO_2_* end-tidal CO_2_ partial pressure; *AV* automated ventilation | | | | | |

**Supplemental content – 5.** Nurse/physician interventions during the daily nursing procedure (*DNP*) according to ventilation mode (*CV* conventional ventilation; *AV* automated ventilation)

| Intervention | CV period  n = 265 | AV period  n = 265 | p |
| --- | --- | --- | --- |
| PEEP increase ^*^ – n (%) | 2 (1) | 53 (20) | < 0.001 |
| Ventilation mode change – n (%) | 1 (0.5) | 1 (0.5) | 1.00 |
| Need for manual ventilation – n (%) | 2 (0.8) | 0 (0) | 0.50 |
| Manual activation of oxygen bypass |  |  |  |
| Number of patients – n (%) | 69 (26) | 41 (15) | 0.004 |
| Number per DNP | 1 ± 1 (1 – 1) | 1 ± 1 (1 – 1) | 0.82 |
| Endotracheal suctioning |  |  |  |
| Number of patients – n (%) | 106 (40) | 89 (34) | 0.15 |
| Number per DNP | 1 ± 1 (1 – 1) | 1 ± 0 (1 – 1) | 0.03 |
| Categorical variables are expressed as n (%) and continuous variable as mean ± standard deviation (95% confidence interval). *PEEP* positive end-expiratory pressure.  ^*^ The PEEP setting was always increased manually during the CV period and automatically by the ventilator during the AV period. | | | |
